# Integral Role of Water in the Solid-State Behavior of the Antileishmanial Drug Miltefosine

**DOI:** 10.1021/acs.cgd.2c00843

**Published:** 2022-09-20

**Authors:** Amy V. Hall, Isobel E. F. Gostick, Dmitry S. Yufit, Gloria Y. Marchant, Preyanthiny Kirubakaran, Shadrack J. Madu, Mingzhong Li, Patrick G. Steel, Jonathan W. Steed

**Affiliations:** † Department of Chemistry, Durham University, Lower Mountjoy, Stockton Road, Durham DH1 3LE, U.K.; ‡ School of Pharmacy, De Montfort University, The Gateway, Leicester LE1 9BH, U.K.

## Abstract

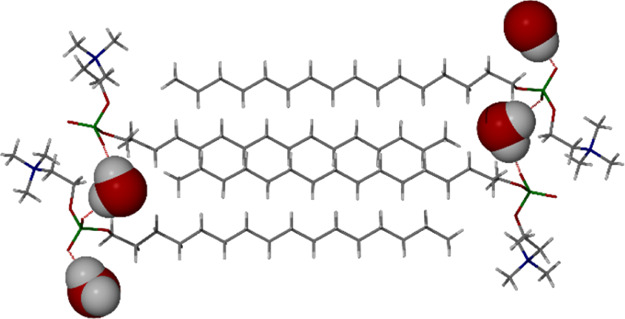

Miltefosine is a repurposed anticancer drug and currently the only orally administered drug approved to treat the neglected tropical disease leishmaniasis. Miltefosine is hygroscopic and must be stored at subzero temperatures. In this work, we report the X-ray structures of miltefosine monohydrate and methanol solvate, along with 12- and 14-carbon chain analogue hydrates and a solvate. The three hydrates are all isostructural and are conformational isomorphs with *Z*′ = 2. Water bridges the gap between phosphocholine head groups caused by the interdigitated bilayer structure. The two methanol solvates are also mutually isostructural with the head groups adopting a more extended conformation. Again, the solvent bridges the gap between head groups in the bilayer. No anhydrous form of miltefosine or its analogues were isolated, with dehydration resulting in significantly reduced crystallinity. This arises as a result of the integral role that hydrogen-bond donors (in the form of water or solvent molecules) play in the stability of the zwitterionic structures.

## Abstract 2

Hydrogen-bond donors in the form of water or solvent molecules play a key role in the stability of the antileishmaniasis drug miltefosine.

## Introduction

**Figure 1:**
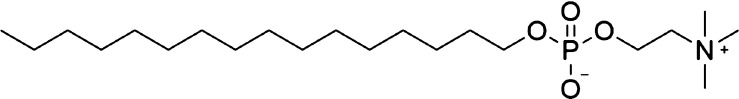
Zwitterionic structure of miltefosine.

Leishmaniasis is a neglected disease that is endemic in the tropics, subtropics, and Mediterranean basin.^1^ The disease is caused by protozoan parasites of the genus *Leishmania* and is transmitted to humans by infected female phlebotomine sandflies.^2^ There are three main manifestations of leishmaniasis: visceral, cutaneous, and mucocutaneous, with visceral leishmaniasis accounting for the most fatalities if left untreated.^3^ There are four different medicines specified on the 22nd list of WHO Model List of Essential Medicines as treatments for leishmaniasis: amphotericin B, pentavalent antimonials, paromomycin, and miltefosine.^4^ Miltefosine is the first and only oral medication to be successfully utilized as a treatment for visceral leishmaniasis but is teratogenic and causes toxicity due to the amphiphilic and zwitterionic structure of the drug (Figure 1) which irritates the gastrointestinal epithelial lining.^5,6^

Miltefosine is hygroscopic,^7^ which suggests it is most stable when surrounded by water molecules. There are many studies of miltefosine at the air/water interface^8,9^ as micelles^10,11^ and as liquid crystals;^12^ however, the studies focusing on the solid-state structure of miltefosine are limited.^13^ Hydrates or solvates can be undesirable in pharmaceutical formulation. For example, hydrate forms of active pharmaceutical ingredients can cause problems for the storage and shelf life of the drug if dehydration takes place. Water can also cause reaction with other excipients within the tablets.^14,15^ Therefore, in this work, we investigate the structure of miltefosine and structural analogues (14- and 12-carbon alkyl chain analogues) with biological activity^6^ to understand the role of water in the materials and determine whether it is possible to prepare an anhydrous form.

## Results and Discussion

### Solid Forms of Miltefosine and Its Analogues

Miltefosine or *n*-hexadecylphosphocholine (PC16) is a white, hygroscopic crystalline powder and is stored at −20 °C and is readily soluble in aqueous and organic solvents.^7^ Fourier transform infrared spectroscopy (FTIR) and thermogravimetric analysis (TGA) characterization of commercial samples of PC16 demonstrate that it is a monohydrate^16−19^ (Figure S1). No single-crystal X-ray diffraction (SC-XRD) structures of PC16 or closely related analogues with different alkyl chain lengths are currently reported in the Cambridge Structural Database (CSD);^20^ however, a related, chiral glycerol-derived phosphocholine structure with an 18-carbon alkyl chain (3-octadecyl-2-methyl-d-*glycero*-1-phosphocholine) is known and also exists as a monohydrate (refcode: DONZAH), suggesting that there may be a consistent structural reason for hydrate formation in this class of compound.^21^ In DONZAH, the molecules pack in a bilayer with a herringbone pattern with interdigitating head groups and hydrocarbon chains, alongside hydrogen bonds from the water molecule to the phosphate oxygen atom at an O···P distance of 2.80 Å and a *Z*′ of 1.^21^

**Figure 2:**
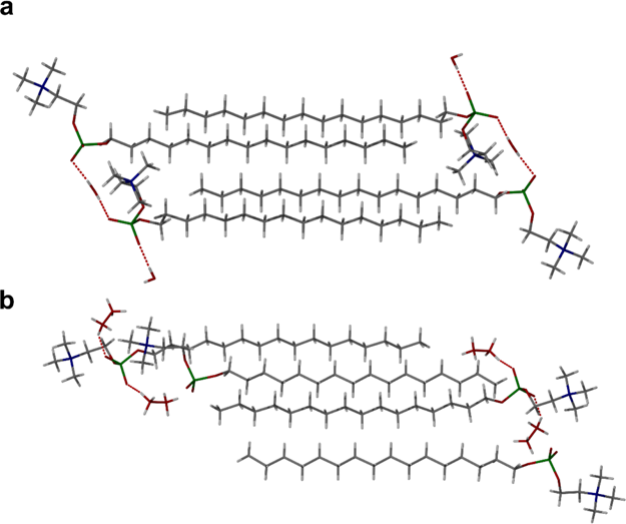
SC-XRD structures of the bilayer arrangement of (a) PC16 monohydrate and (b) PC16 (disordered) methanol solvate.

To obtain the crystals of PC16, a sample was dissolved in 0.5 mL of chloroform and toluene (1:1) and was sonicated at 70 °C for 1 min and allowed to cool to room temperature, which yielded colorless birefringent block crystals after 3 weeks. The SC-XRD determination revealed a centrosymmetric triclinic structure (*P*1̅) with two crystallographically independent molecules of miltefosine and two water molecules. The structure is thus a monohydrate with *Z*′ = 2 (Figure 2a). The water molecule acts as a hydrogen-bond bridge between the phosphocholine headgroups of the two miltefosine molecules, which adopt different head group conformations. The structure is thus a conformational isomorph.^22,23^ This observation further demonstrates the conformational flexibility of this class of molecule.^24,25^ The aliphatic chains interdigitate in the structure, and the role of water seems to be to bridge the distance across the width of the interdigitated C_16_ groups. The hydrogen-bonded chain created by the presence of the water molecules exhibits OH···O distances of 2.805(6) and 2.812(6) Å from the hydroxy group of water to the phosphate oxygen atom of the PC16 headgroup.

A separate crystallization experiment of PC16 resulted in the formation of colorless needle crystals from the slow cooling of 2-butanol after 3 weeks. The SC-XRD analysis revealed a (disordered) methanol solvate of PC16. Methanol appears to arise from inadvertent vapor diffusion from adjacent samples. This PC16 solvate is also a *Z*′ = 2 conformational isomorph with a bilayer structure, but the head group of miltefosine adopts a more extended conformation, resulting in a significantly longer *c* unit cell axis (27.6 vs 24.2 Å), as shown in Figure 2b. This appears to arise from the single hydrogen bond donated by methanol and the larger size of methanol compared to that of water, preventing a hydrogen-bonded chain structure. The disordered methanol molecules hydrogen bond to the phosphate group of PC16 (OH···O), with hydrogen bond distances of 2.581(7) and 2.666(6) Å.

Both of these miltefosine structures imply that the presence of a solvent molecule is necessary to fill gaps between the polar groups left by the bilayer structure. In order to probe the generality of hydrate and solvate formation in this class of compound, we examined the structures of closely related analogues of PC16. We also explored the possibility of producing an anhydrous form of PC16 miltefosine itself.

**Figure 3:**
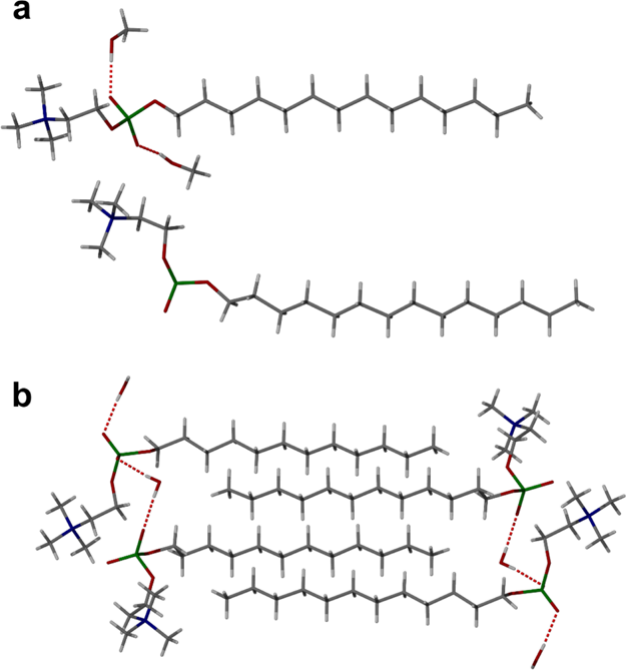
SC-XRD structures of the asymmetric unit of (a) PC14 solvate showing the two different conformers and ordered methanol hydrogen bonding to just one of the PC14 molecules and (b) PC12 hydrate bilayer packing. One of the PC*n* molecules in the asymmetric unit of both the PC14 methanol solvate and the PC12 hydrate structures is disordered over two positions.

In addition to PC16, the SC-XRD structures of two shorter-chain analogues were also determined, namely, *n*-tetradecylphosphocholine (PC14) and *n*-dodecylphosphocholine (PC12). PC14 also crystallizes as both a hydrate and a methanol solvate (Figure 3a), from slow-cooling crystallizations in undried acetonitrile, in the presence of methanol in the latter case. The PC14 hydrate is isostructural to the PC16 hydrate (*Z*′ = 2) with a shorter *c* axis reflecting the shorter alkyl chain, and again water plays an integral role in holding the PC14 molecules together in a hydrogen-bonded chain that spans the width of the interdigitated bilayer. Hydrogen-bonded OH···O distances are 2.760(4) and 2.808(3) Å. The PC14 methanol solvate is also isostructural to its PC16 analogue (a conformational isomorph with *Z*′ = 2) with the same more extended conformations, although in this case most of the methanol is ordered, while one of the PC14 molecules in the asymmetric unit is twofold disordered. Two ordered methanol molecules are situated in discrete pockets hydrogen bonding to two of the phosphate oxygen atoms of just one of the two PC14 molecules which has a more extended conformation, with OH···O distances of 2.685(4) and 2.707(4) Å. The other PC14 molecule has a more compact conformation and accepts a hydrogen bond from a further disordered methanol molecule. In addition, there is a small lattice void that is occupied by an additional partially occupied methanol molecule.

The short-chain analogue PC12 was slowly cooled in acetonitrile and yielded plate crystals after 5 days. The SC-XRD structure reveals that this material is also isostructural to the PC16 and PC14 monohydrates with the same two crystallographically independent conformations and water playing the same head group spanning role (Figure 3b), with OH···O distances of 2.785(3) and 2.817(3) Å. In this case, however, one of the PC12 molecules is disordered across two positions.

### Dehydration Studies

In an attempt to find an anhydrous form of PC16, a range of dehydration, recrystallization, and desolvation studies were undertaken under various conditions. The results were monitored by X-ray powder diffraction (XRPD) and differential scanning calorimetry (DSC). The DSC thermogram of miltefosine monohydrate shows a dehydration endotherm with an onset temperature of 97.8 °C, accompanied by a mass loss of 3.43% by TGA at the same temperature corresponding to 0.8 water molecules for formula unit. This substoichiometric value may represent some empty sites in the crystals.^26^ There are no further changes shown by DSC or TGA until a melt-decomposition endotherm forms, onset 265.4 °C, in agreement with a previous report.^27^ Dehydration was monitored by XRPD. A sample of miltefosine monohydrate was placed on a watch glass and exposed to 120 °C in an oven for 12–120 h. The XRPD pattern at each interval shows a reduction in crystallinity evidenced by considerable peak broadening (Figure S2). While an additional broad, low-angle peak appears at 2.6° 2θ from 24 h drying onward, which might indicate the formation of a material with a larger unit cell, samples exposed proved to be sticky with a tendency to agglomerate, indicating the dried material is highly hygroscopic. These dehydrated samples were analyzed by DSC (Figure S3). The dehydrated samples exhibit evidence for a glass transition at about 55 °C, followed in some cases by a crystallization exotherm. All samples showed two further broad endotherms at lower temperature than the original monohydrate implying desolvation and melt-decomposition of the material of lower crystallinity. This study reveals that after dehydration, the material becomes predominantly amorphous and implies that the water molecules within PC16 hydrate are an integral structural feature of the crystal packing arrangement. This is likely to arise from the lack of hydrogen-bond donors in PC16 itself and hence the inability of the structure to stabilize the polar phosphate groups and span the breadth of the interdigitated bilayer arrangement.

**Figure 4:**
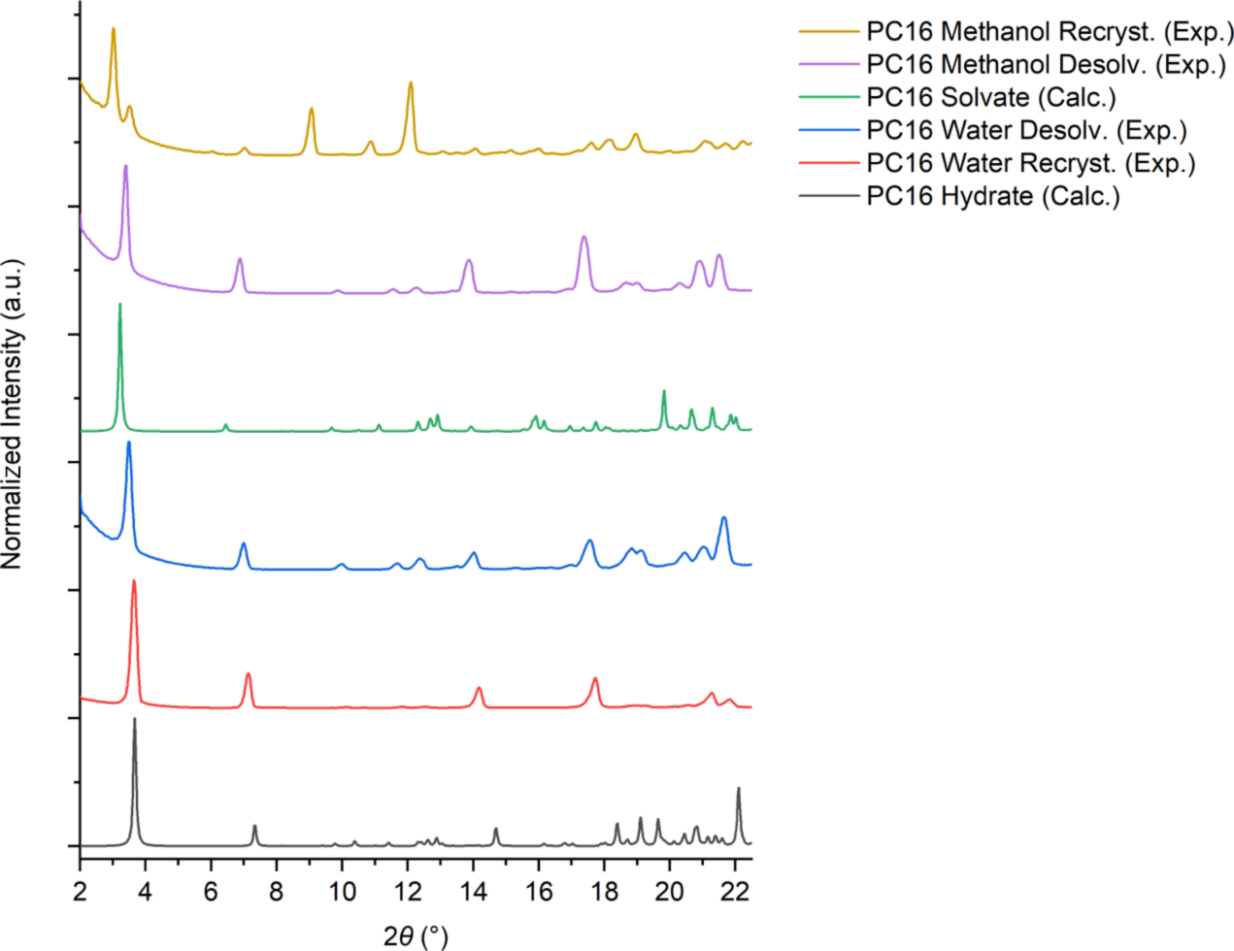
XRPD patterns of PC16 hydrate and PC16 solvate calculated from the SC-XRD data and the experimental patterns of PC16 hydrate and solvate recrystallized in water methanol, respectively. The recrystallized material was then desolvated.

Powdered PC16 hydrate was recrystallized from both water and methanol and analyzed by XRPD and DSC. A comparison of the XRPD patterns of the calculated and experimental powder patterns for the resulting PC16 hydrate and methanolate is shown in Figure 4. The experimental patterns reproduced the calculated patterns well, although some transformation of the methanolate to the hydrate by desolvation and moisture absorption appears to occur on standing. The DSC thermograms of recrystallized PC16 hydrate and solvate indicate the samples are PC16 hydrate only.

The PC16 samples recrystallized from water and methanol were then desolvated by being kept in an oven for 24 h at 60 °C. The XRPD patterns reveal no change in the case of the monohydrate and transformation of the methanolate to the monohydrate structure by adsorption of atmospheric moisture during sample handling.

## Conclusions

The SC-XRD structures of PC16 and other biologically active analogues (PC14 and PC12) demonstrate their tendency to crystallize as an isostructural series of monohydrates or methanolates. Hydrate formation is also observed in the case of the more bulky 3-octadecyl-2-methyl-d-*glycero*-1-phosphocholine, even though the d-glycero substituent significantly alters the packing arrangement.^21^ The conformation of the head group of the PC*n* molecules is dictated by the hydrogen-bonding nature of the solvent, and the interdigitated bilayer structure is retained throughout. Each structure is a conformational isomorph with a *Z*′ value of 2. Dehydration studies of PC16 hydrate reveal that the dehydrated material is of low crystallinity and is unstable and readily reforms the hydrate. Water or methanol acts as an integral part of the structure bridging the bilayer breadth and stabilizing the strong hydrogen-bond-acceptor phosphate groups.

## Experimental Section

### General

All reagents and solvents were purchased from standard commercial sources and used without further purification. FTIR was carried out using a PerkinElmer Spectrum 100 spectrometer, fitted with a diamond universal attenuated total reflectance accessory. Eight scans were collected for each sample at a resolution of 2 cm^–1^ over a wavenumber region of 4000–500 cm^–1^. DSC studies were carried out using a NETZSCH DSC 214 Polyma (NETZSCH instrument, Wolverhampton, UK) operated with nitrogen gas. Samples (approx. 4–6 mg) were weighed in an aluminum pan and hermetically sealed, and the lid was pierced. Samples were then heated at 20 °C min^–1^ from 20 to 300 °C. TGA was carried out by Ruston Services using a TA Instruments Q 500 TGA analyzer. Between 1 and 5 mg of the sample was weighed in platinum pans, and dry nitrogen was used as the purge gas (flow rate: 60 mL min^–1^). XRPD patterns were recorded on glass slides using a Bruker AXS D8 ADVANCE diffractometer with a Lynxeye Soller PSD detector or a Bruker D2 phaser diffractometer equipped with a LYNXEYE XE-T detector using Cu Kα radiation at a wavelength of 1.5406 Å. Single-crystal structures were collected at 120 K using the Bruker D8 Venture diffractometers Photon III MM C14 or C7 CPAD detector, IμS- or IμS-III-microsource, focusing mirrors; λMoKα radiation (λ = 0.71073 Å) equipped with Cryostream (Oxford Cryostreams) open-flow nitrogen cryostats. All structures were solved using direct methods and refined by full-matrix least squares on *F*^2^ for all data using SHELXL^28^ and OLEX2^29^ software. All nondisordered nonhydrogen atoms were refined with anisotropic displacement parameters; disordered atoms in structures PC14 (solvate) and PC16 (solvate) were refined with fixed equal occupancies. CH hydrogen atoms were placed in calculated positions and assigned an isotropic displacement factor that is a multiple of the parent carbon atom and allowed to ride. H atoms attached to oxygen atoms were located on the difference map where possible or placed in calculated positions. Crystallographic data for the structures have been deposited with the Cambridge Crystallographic Data Centre as supplementary publication CCDC 2192420—2192424.

### PC16 Hydrate

Miltefosine (*n*-hexadecylphosphocholine, PC16) is a white crystalline powder supplied in its hydrated form. Analysis calcd for C_21_H_48_NO_5_P: C, 59.26; H, 11.37; and N, 3.29%. Found: C, 58.98; H, 11.23; and N, 3.32%. Crystals of PC16 hydrate were obtained by dissolving PC16 (0.0050 g, 0.012 mmol) in chloroform/toluene (1:1, 0.5 mL), shaking the sealed vessel, and sonicating at 70 °C for 1 min and cooling to room temperature, which yielded colorless birefringent block crystals after 3 weeks. Crystal data: *M* = 425.57 g mol^–1^, triclinic, space group *P*1̅ (no. 2), *a* = 9.4657(7) Å, *b* = 10.8513(8) Å, *c* = 24.2121(18) Å, α = 90.460(2)°, β = 94.964(2)°, γ = 91.296(2)°, *V* = 2476.9(3) Å^3^, *Z* = 4, *D*_c_ = 1.141 g/cm^3^, μ = 0.139 mm^–1^, *F*(000) = 944.0, 42928 reflections collected, 10780 unique (*R*_int_ = 0.0692). Final GooF = 1.051, *R*_1_ = 0.0626 [7665 reflections with *I* ≥ 2σ(*I*)], w*R*_2_ = 0.1317 (all data), 529 parameters, 0 restraints.

### PC16 Solvate

Miltefosine (*n*-hexadecylphosphocholine, PC16, 0.0050 g, 0.012 mmol) was combined with 2-butanol (0.30 mL), heated to 95 °C, sealed and shaken, cooled to room temperature, and reheated to 95 °C. The resulting colorless solution was left to cool slowly, which yielded colorless needle crystals of a miltefosine methanol solvate after 3 weeks. The sample was inadvertently exposed to methanol in the laboratory. Solvent molecules are disordered. Crystal data: *M* = 447.61 g/mol, triclinic, space group *P*1̅ (no. 2), *a* = 8.6690(9) Å, *b* = 10.9861(13) Å, *c* = 27.648(3) Å, α = 95.027(5)°, β = 95.074(4)°, γ = 96.828(4)°, *V* = 2591.4(5) Å^3^, *Z* = 4, *D*_c_ = 1.147 g/cm^3^, μ = 0.137 mm^–1^, *F*(000) = 994.0, 24643 reflections collected, 9812 unique (*R*_int_ = 0.1166). Final GooF = 1.032, *R*_1_ = 0.0976 [4625 reflections with *I* ≥ 2σ(*I*)], w*R*_2_ = 0.2618 (all data), 515 parameters, 0 restraints.

### PC14 Hydrate

*n*-Tetradecylphosphocholine (PC14) is a white crystalline powder and is supplied as a hydrate. Analysis calcd for C_19_H_44_NO_5_P: C, 57.40; H, 11.16; and N, 3.52%. Found: C, 57.46; H, 11.03; and N, 3.45%. Acetonitrile (0.80 mL) was added to *n*-tetradecylphosphocholine (0.0050 g, 0.013 mmol), heated to 80 °C, sealed and shaken, cooled to room temperature, and then heated to 80 °C. The sealed vessel was allowed to cool slowly, yielding colorless birefringent plate crystals of *n*-tetradecylphosphocholine monohydrate after 1 week. Crystal data: *M* = 397.52 g/mol, triclinic, space group *P*1̅ (no. 2), *a* = 9.4389(12) Å, *b* = 10.7939(13) Å, *c* = 22.371(3) Å, α = 92.010(4)°, β = 90.886(4)°, γ = 91.099(4)°, *V* = 2277.1(5) Å^3^, *Z* = 4, *D*_c_ = 1.160 g/cm^3^, μ = 0.147 mm^–1^, *F*(000) = 880.0, 44906 reflections collected, 9933 unique (*R*_int_ = 0.1542). Final GooF = 1.007, *R*_1_ = 0.0737 [4573 reflections with *I* ≥ 2σ(*I*)], w*R*_2_ = 0.1914 (all data), 484 parameters, 0 restraints.

### PC14 Solvate

*n*-Tetradecylphosphocholine (PC14) methanol solvate was prepared as the result of a failed solution cocrystallization of PC14 and t-butylhydroquinone in a 1:2 ratio, respectively. PC14 and t-butylhydroquinone were combined with acetonitrile (0.50 mL), heated to 80 °C, sealed, shaken, and cooled to room temperature. Methanol (0.10 mL) was then added, and the mixture was heated to 60 °C before sealing, shaking, and leaving to cool slowly. Colorless translucent prism-shaped crystals were yielded after 4 weeks and were found to be a disordered methanol solvate of PC14 in a 1:2.5 ratio. Crystal data: *M* = 418.55 g/mol, space group *P*1̅ (no. 2), *a* = 8.6781(6) Å, *b* = 10.9554(7) Å, *c* = 25.0960(16) Å, α = 99.656(2)°, β = 97.428(3)°, γ = 95.037(2)°, *V* = 2317.7(3) Å^3^, *Z* = 4, *D*_c_ = 1.199 g/cm^3^, μ = 0.149 mm^–1^, *F*(000) = 926.0, 52057 reflections collected, 12261 unique (*R*_int_ = 0.0492). Final GooF = 1.074, *R*_1_ = 0.1036 [9765 reflections with *I* ≥ 2σ(I)], w*R*_2_ = 0.2896 (all data), 658 parameters, 46 restraints.

### PC12 Hydrate

*n*-Dodecylphosphocholine (PC12) is a white crystalline powder and is supplied in its hydrated form. Analysis calcd for C_17_H_40_NO_5_P: C, 55.26; H, 10.91; and N, 3.79%. Found: C, 55.68; H, 10.80; and N, 3.67%. Acetonitrile (0.70 mL) was added to *n*-dodecylphosphocholine (0.0050 g, 0.014 mmol), heated to 80 °C, sealed and shaken, cooled to room temperature, and then heated to 80 °C. The sealed vessel was allowed to cool slowly, yielding colorless birefringent plate crystals of *n*-dodecylphosphocholine monohydrate after 5 days. Crystal data: *M* = 369.47 g/mol,, triclinic, space group *P*1̅ (no. 2), *a* = 9.4601(7) Å, *b* = 10.7497(7) Å, *c* = 20.9618(14) Å, α = 81.907(2)°, β = 86.909(3)°, γ = 89.774(2)°, *V* = 2107.4(3) Å^3^, *Z* = 4, *D*_c_ = 1.165 g/cm^3^, μ = 0.154 mm^–1^, *F*(000) = 816.0, 40972 reflections collected, 11166 unique (*R*_int_ = 0.0811). Final GooF = 1.032, *R*_1_ = 0.0730 [7489 reflections with *I* ≥ 2σ(I)], w*R*_2_ = 0.2012 (all data), 547 parameters, 0 restraints.

## Supporting Information Available

IR spectra, XRPD diffractograms, and DSC data and crystal structure deposited with the Cambridge Structural Database CCDC 2192420—2192424 (PDF)
