# Supplementary material for: Integral Role of Water in the Solid-State Behavior of the Antileishmanial Drug Miltefosine
Source: Cryst Growth Des. 2022 Sep 20;22(10):6262–6. doi: 10.1021/acs.cgd.2c00843 (PMC9542694; doi:10.1021/acs.cgd.2c00843)
Supplement: Supplementary file 1 — cg2c00843_si_001.pdf [file cg2c00843_si_001.pdf]

The Integral Role of Water in the Solid-State Behaviour of the Antileishmanial Drug Miltefosine

Amy V. Hall,[a] Isobel E. F. Gostick,[a] Dmitry S. Yufit,[a] Gloria Y. Marchant,[a] Preyanthiny Kirubakaran,[b] Shadrack J. Madu,[b] Mingzhong Li,[b*] Patrick G. Steel,[a] and Jonathan W. Steed.[a*]

a) Durham University, Department of Chemistry, Lower Mountjoy, Stockton Road, Durham, DH1 3LE, UK. E-mail: jon.steed@durham.ac.uk
b) De Montfort University, School of Pharmacy, The Gateway, Leicester, LE1 9BH.

Supplementary Information

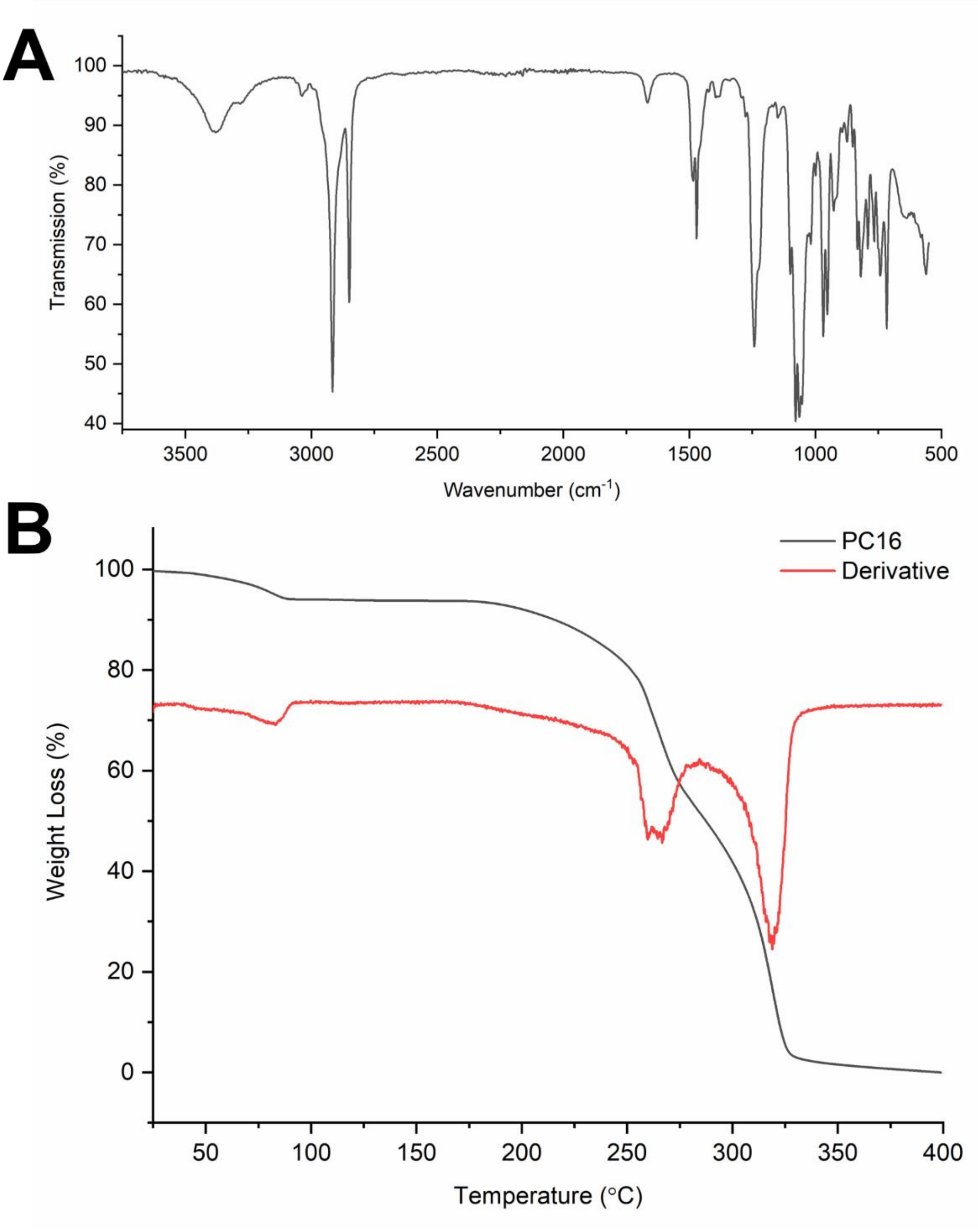

Figure S1. The broad O-H band in the FTIR spectrum between 3500 and 3000 cm^{-1} (A), and the weight loss that occurs with an onset temperature of 71 °C in the TGA thermogram (B) suggests the presence of water in PC16 as supplied.

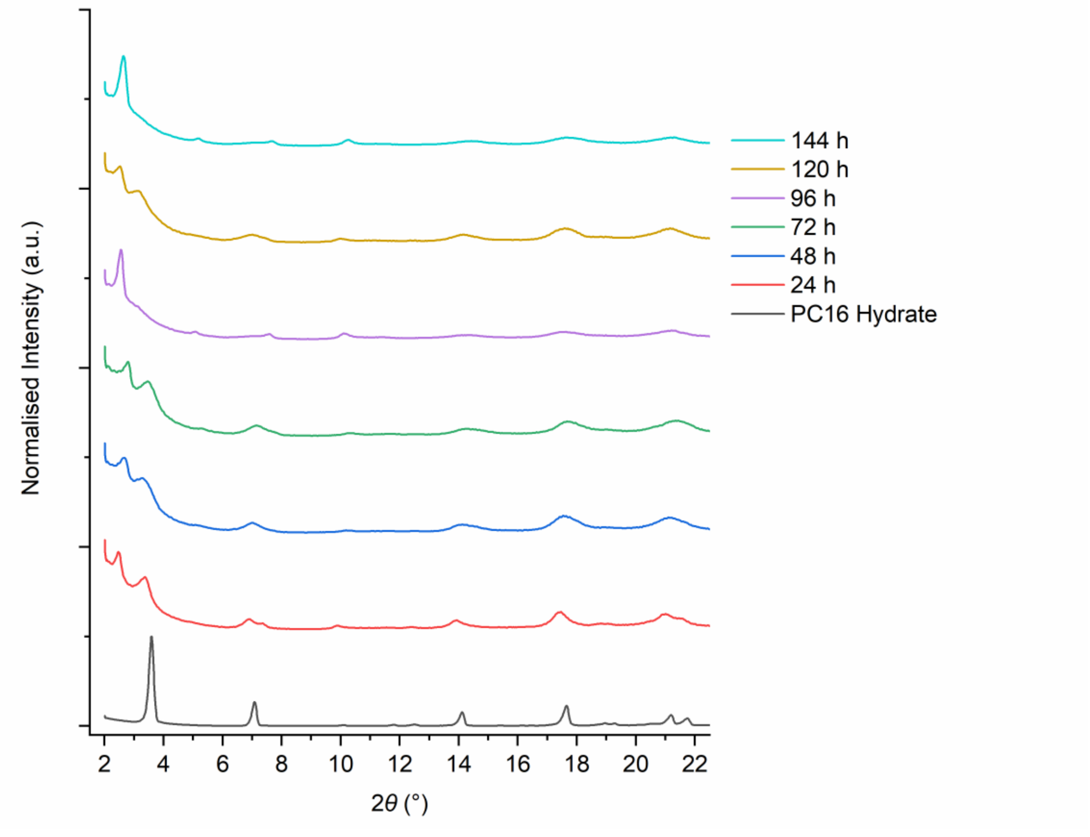

Figure S2. The XRPD patterns of PC16 hydrate exposed to 120 °C for different durations.

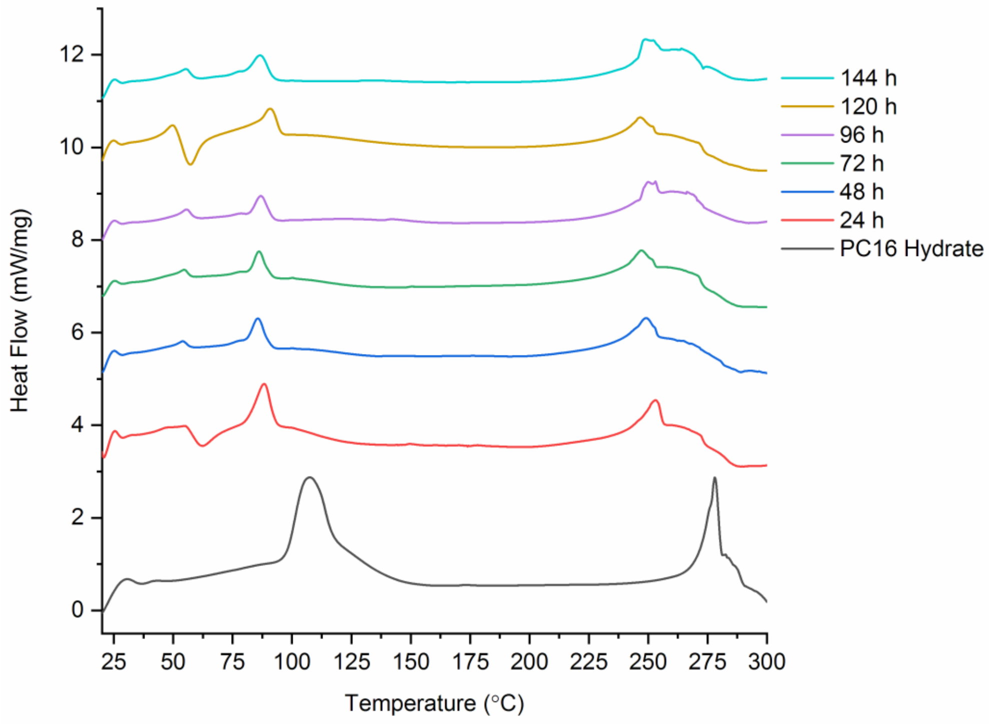

Figure S3. The DSC thermogram of PC16 hydrate before and after heating to 120 °C for durations between 24-144 hours.
